# Position Measurement/Tracking Comparison of the Instrumentation in a Droplet-Actuated-Robotic Platform

**DOI:** 10.3390/s130505857

**Published:** 2013-05-07

**Authors:** Renaud Casier, Cyrille Lenders, Marion Sausse Lhernould, Michaël Gauthier, Pierre Lambert

**Affiliations:** 1 BEAMS Department, CP 165/56, Université Libre de Bruxelles, 50 Avenue FD Roosevelt, Brussels B-1050, Belgium; E-Mails: renaud.casier@ulb.ac.be (R.C.); cyrille.lenders@ulb.ac.be (C.L.); marion.sausse@ulb.ac.be (M.S.L.); 2 FEMTO-ST/CNRS, 24 rue Alain Savary, Besançon F-25000, France; E-Mail: michael.gauthier@femto-st.fr

**Keywords:** micro-assembly, microfluidics, drops, bubbles, positioning

## Abstract

This paper reports our work on developing a surface tension actuated micro-robotic platform supported by three bubbles (liquid environment) or droplets (gaseous environment). The actuation principle relies on the force developed by surface tension below a millimeter, which benefits from scaling laws, and is used to actuate this new type of compliant robot. By separately controlling the pressure inside each bubble, three degrees of freedom can be actuated. We investigated three sensing solutions to measure the platform attitude in real-time (z-position of each droplet, leading to the knowledge of the z position and Θ_x_ and Θ_y_ tilts of the platform). The comparison between optical, resistive, and capacitive measurement principles is hereafter reported. The optical technique uses SFH-9201 components. The resistive technique involves measuring the electrical resistance of a path flowing through two droplets and the platform. This innovative technique for sensing table position combines three pairs of resistances, from which the resistance in each drop can be deduced, thus determining the platform position. The third solution is a more usual high frequency (∼200 MHz) capacitive measurement. The resistive method has been proven reliable and is simple to implement. This work opens perspectives toward an interesting sensing solution for micro-robotic platforms.

## 1. Introduction

Micro-assembly deals with the assembly of submillimeter components. Operations that grip, move, place, and release microcomponents to defined locations have to deal with forces inherent to the microworld. To minimize the effects of some of these forces, which are difficult to control, one strategy involves performing the manipulation in a liquid [1]. Consequently, we developed a microrobotic platform to perform assembly operations that is based on the use of the surface tension effect of gaseous bridges (droplets) between two solids in a liquid. Such a table could be used as a support for assembly operations, with a moving microgripper transporting and assembling new components [2]. Moreover, assembling micro-components requires introducing compliance into the system to avoid high stress in fragile materials caused by positioning or manufacturing errors. A solution that relies on the elastic compliance of springs has already been presented by Clévy [3]. In our design, the use of bubble and drops as actuators introduces a new compliance source due to the elasticity of the liquid-gas interface (surface tension effect). This leads to six degrees of freedom, three of which are controlled by the pressure in the drops or bubbles. Evaluating the table position (*i.e.*, these three actuated degrees of freedom) is the topic of this paper. Section 2 therefore describes the microrobotic platform. We introduce an innovative position sensing technique based on measurements of the electrical resistance of droplets. This method is implemented, tested, and compared to more traditional methods involving photo-sensors [4,5] and capacitive measurements [6]. The main conclusion is that the resistive method is repeatable and precise; moreover, it is simple to implement, cheap, and easy to automate. However, the resistance method's main drawback is that supplementary information is necessary to determine the actuator position using this method, such as the droplet volume, its height, or its shape. The resistance method is discussed in detail below, starting with a description of the microrobotic platform, followed by a theoretical description of the three sensing solutions in Section 3. The three following sections describe and interpret tests performed using photosensors, capacitive measures, and resistive measures. Finally, Section 7 presents a comparison of three sensing solutions and analyzes the results before concluding.

## 2. Description of the Microrobotic Platform

### 2.1. Modeling and Development

**Figure 1.**
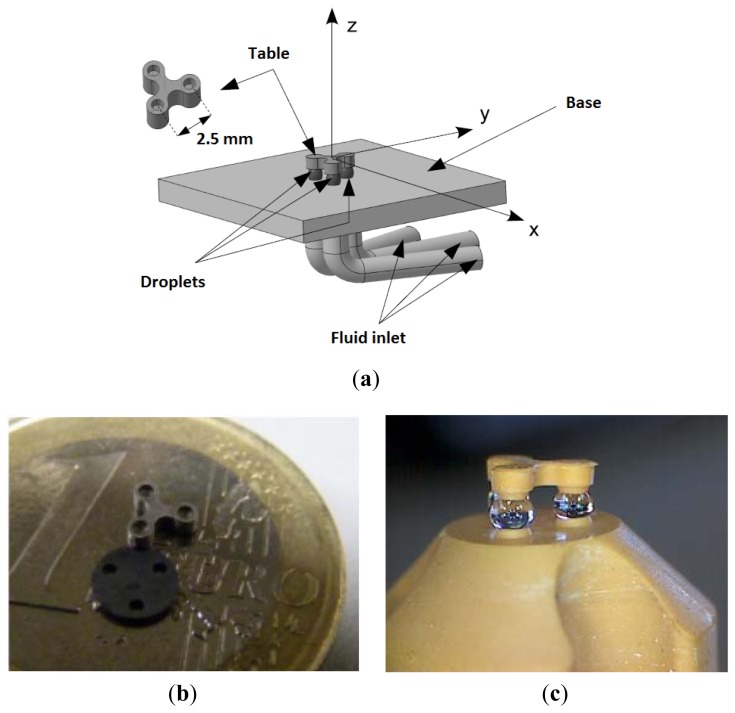
(**a**) General views and principle: The three fluid income pipes control the pressure in the three droplets, acting as z-actuators able to modify the z position and the Θ_x_ and Θ_y_ tilts; (**b**) Scale comparison: The pitch between to droplets seats is 2.5 mm; (**c**) water droplets supporting the platform manufactured by additive manufacturing (Courtesy Marc Viallon, Plateforme Prototypage Microtechniques, Lycée Edgar Faure, 25500 Morteau, France).

The device presented (Figure 1) here is a compliant platform that can be used to perform microrobotic assembly tasks in a liquid environment [7,8]. The table has six degrees of freedom (DOFs), of which three are actuated: the translation along the direction orthogonal to the platform plane z, and two rotations along axes parallel to the platform plane.

The device is made out of three main components: a moving table, three droplets, which are used as compliant actuators, and the platform from which the droplets are generated. Thanks to surface tension at the gas-liquid interface, and to the gas's compressibility, a microbubble can be used as a compliant actuator. Indeed, the force developed by a bubble sandwiched between two solids has two components: the surface tension along the triple line (where solid, liquid, and gas touch one another) and the pressure gradient across the interface. If the contact angles in the liquid are smaller than 90°, the mean curvature of the bubble interface is positive, and the pressure inside the bubble is larger than in the surrounding liquid. When the bubble is squeezed, it is compressed and exerts a repulsive force on the solids. When it is stretched, it exerts an attractive force on the solids. Its behavior is comparable to a spring, from a mechanical point of view. A bubble presents several advantages compared with classical mechanical springs, for example, the possibility to actuate droplets using the fluidic parameters pressure P and volume V. We use a system derived from a syringe pump to control bubble generation and thus actuate by varying the volume of a tank containing gas in order to push the gas out of a hole. This principle offers the ability to generate a single bubble at a specific location and to easily control its shape via mechanical control (the position of the piston in the syringe).

### 2.2. Realization

The prototype is made out of aluminum and was manufactured using a conventional computer numerical control milling machine [2]. The anchoring was realized using a mechanical method consisting of creating a sudden variation in the solid part profile in order for the contact angle θ to increase up to an angle called the advancing angle value before the triple line can move.

The bubble generator is based on a volume control, like a syringe pump. In the prototype, the bubble generator is a flexible hose containing the gas, which is squeezed to generate the bubble. The volume of gas initially contained in the hose can be adjusted by filling the hose with an incompressible fluid.

Alternatively, the device can also be produced by additive manufacturing, as shown in Figure 1(c). The latter prototype was produced with a Perfactory machine (Envisiontec, Gladbeck, Germany) with a 42 μm lateral resolution and a 50 μm vertical resolution. This production technique is based on the photopolymerization of a resin (further information on rapid prototyping can be found in [9]).

### 2.3. Context

The platform shows very promising characteristics, for example, auto-centering and resilience to vibrations, disturbances, or impacts. These advantages could considerably improve the ability to industrially position and handle micro-components. The working principle is validated [3] and the need shifts to finding an efficient, easily implemented, low-cost means of position sensing for the table. Indeed, optical sensors are typically used but need to be replaced by a system that allows quicker, more precise, automated evaluation and table piloting.

## 3. Theoretical Description of the Three Sensing Solutions

Three points of measure are necessary to completely define the three-dimensional positioning of the table. The table height and orientation can easily be deduced using the exact height of these points compared to the base. While looking for a new measurement method, the prototype was first equipped with high precision optical cameras able to detect the table orientation along the two transversal axes. Unfortunately, this cannot be considered for industrial purposes because cameras are large, fragile, and expensive. Embedded solutions are thus preferable. Three more classical methods are studied: photo-sensors, capacitive measurement, and an innovative method based on resistive measurements in droplets. Studied designs and the experimental protocols validating the methods performances are described hereafter. In these methods, the imposed displacement was also measured with a non contact displacement sensor (Keyence LC-2440 laser, Osaka, Japan) with a resolution of 0.2 micron. The different measurements methods are thus consistent considering the precision of the measurement, and errors are consistent across the 3 methods.

### 3.1. Optical Solution

**Figure 2.**
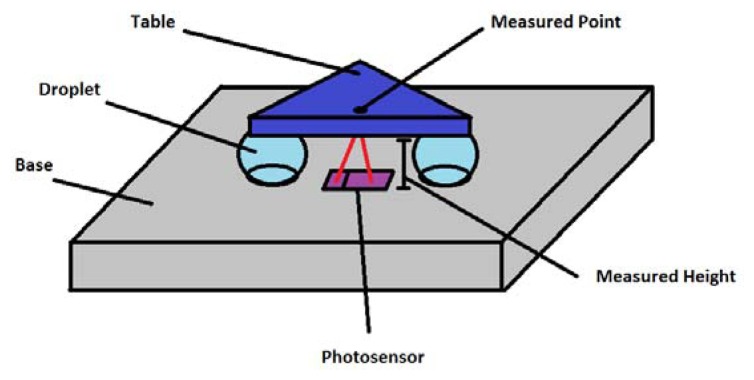
Schematic representation of reflective photo-sensors directed toward the mid-point of each side of the table (full three-dimensional positioning of the table in space needs three points of measure).

The measurement principle is based on the use of a miniaturized reflective photo-sensor, which has four interesting characteristics; it is flat, short-ranged, cheap, and low needs in terms of annex electronics. For geometrical reasons, the photo-electric sensors are placed between two holes in order for the three sensors to be directed toward the midpoint of each side of the table. We tested a SFH-9201 Reflective Interrupter [10], with an optimal operating distance of 1 mm to 5 mm. Its working principle is simple. An infrared emitter glows a cone of infrared light in front of the sensor. This light is reflected by the target and comes back to the sensor (Figure 2). The receptor is a NPN transmitter whose collector current is related to the degree of infrared illumination it is receiving. A simple electronic circuit is then used to measure this current, from which the distance can be deduced.

### 3.2. Electrical Solutions: Resistive and Capacitive Measurements

**Figure 3.**
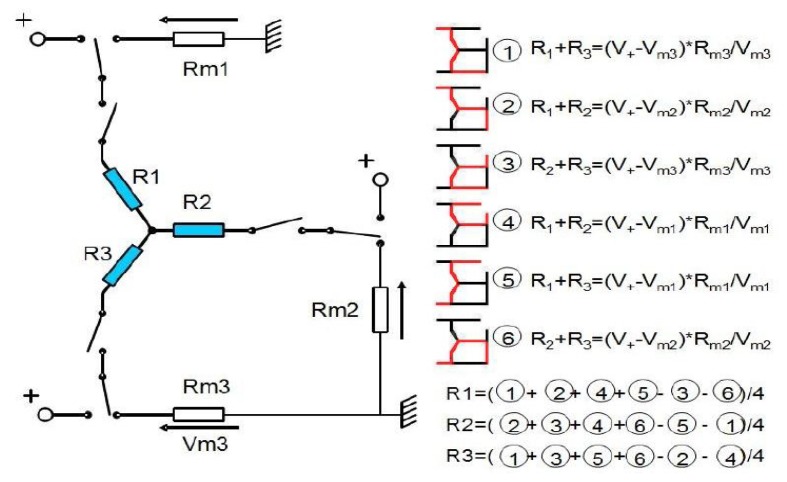
Principle of alternated resistance measure. The six circuits represented are alternatively formed thanks to switches. The displayed equation uses the values of Rmi and the associated tensions Vmi to provide the values of Ri (i = 1,2,3).

Among electrical measures able to give information on the table position is the measure of electrical resistance in droplets. Each bubble is considered individual resistance Ri and the conducting table connects the droplets (in Figure 3, shown below, the table is located at the node connecting R1, R2, and R3). These unknown resistances can be measured using the electrical circuits shown in the right insight of Figure 3, leading to the indicated electrical equations.

If speed frequency from one circuit to the other is fast enough, the sampled resistances will lead to the platform position because there is a link between the resistance and the geometry. Thus, Figure 3 illustrates the equivalent electrical circuit for the bubbles and the table. If the switching frequency is high enough, the resistance of each bubble and droplet can be determined using the following equation:

(1) $$\begin{array}{cc} R=\frac{l.\rho }{S}\;\text{with}\; & \rho_{\text{purewater}}=1,8\times 10^{5}\Omega .m \end{array}$$

where *l* is the length of the conductor, *ρ* the conductivity, and *S* the surface of the conductor. A more correct geometric model of the droplets will be considered in Section 6.

**Figure 4.**
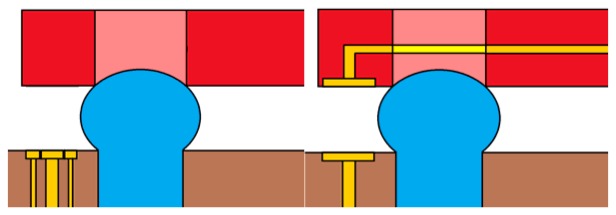
Capacitive probes in configurations using three capacitive probes between each droplet pair. The measure is achieved through an oscillator that is properly calibrated using the capacitance of air under the table as the oscillating capacitor. The frequency exciting the oscillator is then directly related to the capacitance.

The second available electrical method is the capacitive positioning measurement, which is widely validated in industrial applications and literature [6]. Here, we measured the electrical capacitance of the air gap between the table and the base (Figure 4). This solution has the advantage of being independent from the fluidic circuit. Droplets' height *d* can be related to capacitance using the following equation:

(2) $$C=\frac{\varepsilon *S}{d}\wedge \varepsilon =\varepsilon_{r}*\varepsilon_{0}$$

Since S∼1e−6 m^2^, d∼100e−6 m, epsilon = 1, and epsilon 0 = 8.85e−12 F/m, C is approximately 10e−13 F. The voltage frequency must then be large enough to measure the voltage drop across the gap. This process will be detailed in Section 5.

## 4. Photosensors Testing

A test bench composed of a fix support for the chip and a mobile target was designed. The position of the target is adjusted using a millimeter head. It is then recorded and the collector current of the phototransistor is measured thanks to an electronic comparator. During these tests, all reachable positions by the photo-sensor were investigated. Two main parameters were studied: (1) the angle α between the surface of the target and the surface of the sensor, the two scenarios being tested, the parallel target, and a small angle of 5° to 8°; (2) the type of surface of the target, a highly reflective metallic surface and a highly non-reflective mat blank surface. Hereafter are displayed the resulting graphs showing the output voltage of the electronic device measuring the transistor response related to the distance of the target.

**Figure 5.**
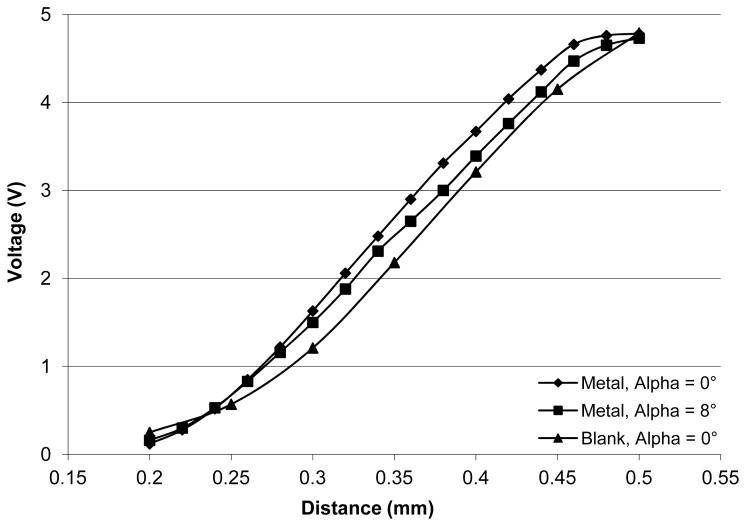
Influence of the tilt angle of the reflective surface on the sensor output. The curve is independent from the tilt angle in the linear region.

Figure 5 shows a series of experimental results focused on the short-range behavior of the sensor. These results expose the error induced by the maximum target angle, that is, eight degrees, on the distance measurements. The distance determination error is lower than 20 μm, meaning lower than 5% of the measured value. In these conditions, and taking into account that the angle used for these experiments is higher than the usual operation status of such a device, the measuring capabilities of this sensor can be considered satisfactory.

Other tests showed excellent repeatability, good sensor positioning, and negligible edge effects. The positive aspects of this sensing solution include low cost and simplicity of use. Its drawbacks are related to size, with a width of 5.8 mm (pins included), while the space between two droplets is only 2.5 mm.

## 5. Resistive Measurements Tests

As already stated in Section 3, droplets' geometry deviates from the cylindrical shape whose resistance is derived though Equation (1). Simulations were therefore performed thanks to the mathematic virtual environment MatLabv.R2010a to take the droplets' geometry into account.

**Figure 6.**
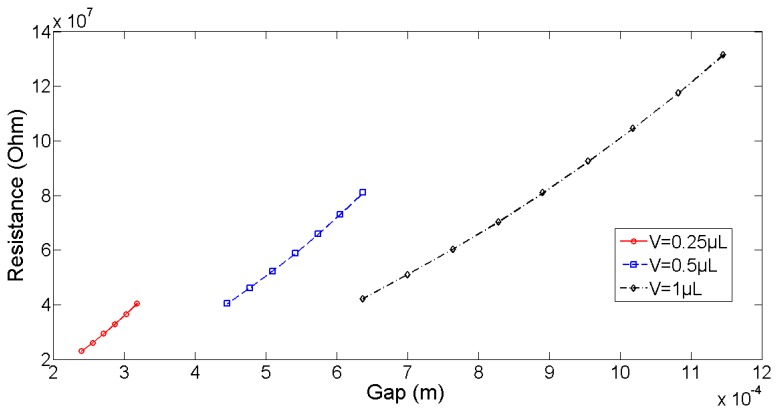
Electrical resistance of a meniscus anchored between two circular pads as a function of the meniscus height for different volumes of liquids. Simulation parameters: pad radius = 0.5 mm, surface tension = 72 mN/m and liquid conductivity = 1e−5 S/m. The almost linear dependence will serve as the measuring principle tested in the next section.

Based on the work developed by Lenders [1], we know that the problem of a meniscus anchored between two circular pads is described by four parameters: the force F applied on the meniscus (one third of the load applied on the platform if applied symmetrically), the capillary pressure in the meniscus (which is related to the meniscus curvature and the surface tension created by the so-called Laplace law), the volume of liquid, and the meniscus gap, h. These parameters are related by a force equation and the Laplace law. The force equation relates F to the geometry, expressed as the sum of the tension force along the contact line and the pressure force. In axisymmetric geometry, the Laplace law can be rewritten into a non-linear second order differential equation with an unknown parameter (*i.e.*, the capillary pressure). This therefore requires that three conditions be solved. Two conditions are provided by the boundary conditions (top radius = bottom radius = pad radius), while the capillary pressure can be adjusted to respect the prescribed volume of liquid (this volume of liquid has therefore to be measured in the experiment, which is done at the beginning as an initial condition. We discuss in the conclusion the assumption of constant volume of liquid throughout the experiment). Therefore, for a given volume of liquid and a given height (which will be the experimental conditions in the next section), the capillary pressure, the force, and the electrical resistance become dependent parameters. Figure 6 represents the electrical resistance of various volumes of liquid as a function of the gap. In these simulations, the pad radius selected was equal to 0.5 mm, the surface tension equal to 72 mN/m, and the liquid conductivity sigma = 1e−5 S/m (theoretical conductivity of DI water). We see that the resistance almost linearly depends on the gap, that this relationship is univoque for a prescribed volume of liquid, and that the slope of this almost linear dependence seems to slightly decrease with increasing volumes of liquids (221 MOhm/mm for V = 0.25 μL, 211 MOhm/mm for V = 0.5 μL and 1.75 MOhm/mm for V = 1 μL).

**Figure 7.**
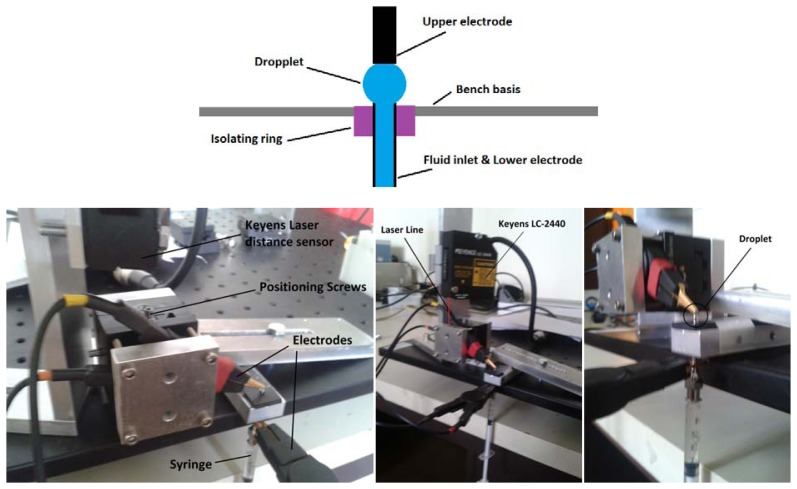
Resistive measurements, measuring electrodes, Keyence laser distance. Sensor & laser line, injection syringe and position of the droplet.

To experimentally measure the electrical resistance of the droplets, a test bench was realized. Simulations confirm that resistance values are very high (∼100 MOhm), which is why a very precise impedance-meter (WAYNE KERR Precise Component Analyzer 6425, Chichester, UK) is used. The measurements of the distance between the bubble base and its top vary between 200 μm and 500 μm. A Keyence LC-2440 laser is used to acquire the necessary resolution for these measurements. A syringe containing distilled water is vertically suspended so that its needle pierces a hydrophobic polymer material. The end of this needle simulates the exit of one of the table basis pipes. One electrode of the impedance-meter is connected to the syringe metallic needle, while the other electrode plays the role of the table, ready to make contact with the droplet as it exits the needle (Figure 7).

**Figure 8.**
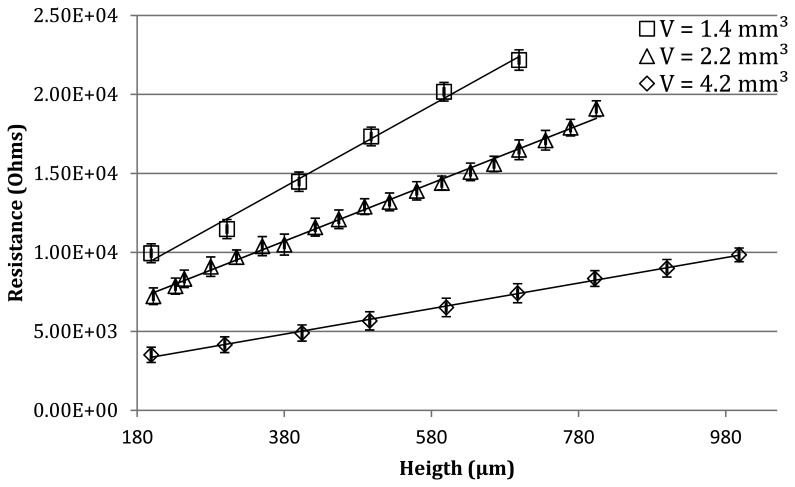
Droplet resistance as a function of its height for increasing volumes. The linear behavior with direction coefficient directed by volume is coherent with the simulations. The average curve showed a standard deviation of 606.9 Ohms, 551.1 Ohms, and 527.1 Ohms, respectively.

The electrical resistance of droplets is measured for the different volumes of water and for different values of the separation distance between the needle from which the bubble emerges and the top electrode. In the first step, the electrode is placed far from the contact position and the droplet is created (for three different volumes). The electrode is then lowered until contact occurs, which represents the zero position. The electrode is then progressively lowered until reaching 200 μm, pressing on and deforming the droplet more and more. At each step, the drop resistance is recorded, and results can be found in Figure 8.

Experimental measurements and theoretical results demonstrate coherent behavior: R increases linearly as a function of H, with the slope decreasing as the volume of liquid increases. Although the quantitative measurements of the resistance show significantly smaller results (about 10 kOhm) than expected from the simulation (about 100 MOhm), this result could be explained by water contamination, which could influence the impedance exhibited by the tests bench. The quasi-linear behavior of the resistance with the drop length is however confirmed. The slope coefficient decreases with volume augmentation. The conclusion is thus similar to what was encountered in theory. The resistive measurements of the droplet height is however impossible without some supplementary information such as the volume of liquid. Indeed, for similar height, droplets with different volumes and shapes could exhibit different electrical resistances. Achieving a positioning system based on resistive measurement thus implies additional features that provide enough information to be able to fully describe the system state. More particularly, for the resistive method to be used, we need secondary information for each droplet. This additional information can be for instance the volume of liquid, which may be part of the initial conditions of the system. In case of evaporation however, this raises further questions.

## 6. Capacitive Measures

**Figure 9.**
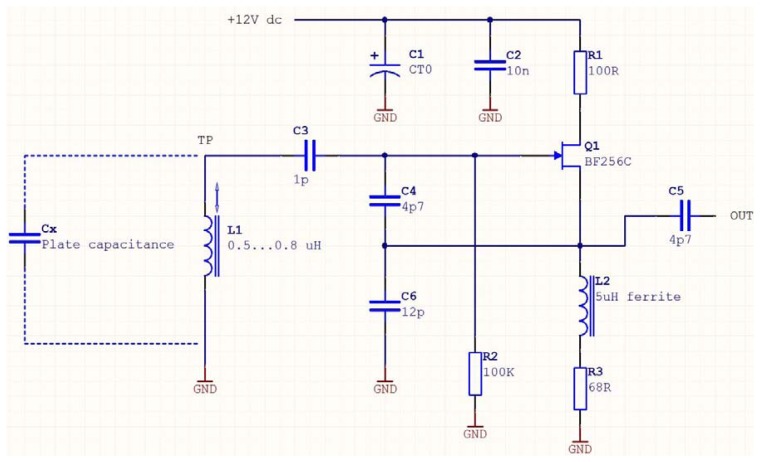
Test oscillator. Cx is the measured capacitance. Output frequency is taken at C5.

Considering that the surface of the electrode is one squared millimeter, the minimum and maximum values of capacitance encountered are, from Equation (3), only a few hundredths of a Pico farad. Typical electronic oscillators have an output frequency inversely proportional to the resonating capacitance, resulting in high frequencies. It was thus important to build an oscillator able to resonate at a sufficiently high frequency. The test oscillator is a Colpitt RF oscillator (active device FET BF256C). Figure 9 illustrates its functioning, with C1 and C2 being decoupling capacitors. The oscillator tank circuit is made of L1 and the three capacitors—C3, C4, and C6—in series. Its oscillation frequency obeys the following rule:

(3) $$F=\frac{1}{2\pi \sqrt{L_{1}\left(\frac{c_{3}c_{4}c_{6}}{c_{3}c_{4}+c_{4}c_{6}+c_{6}c_{3}}+C_{x}\right)}}$$

**Figure 10.**
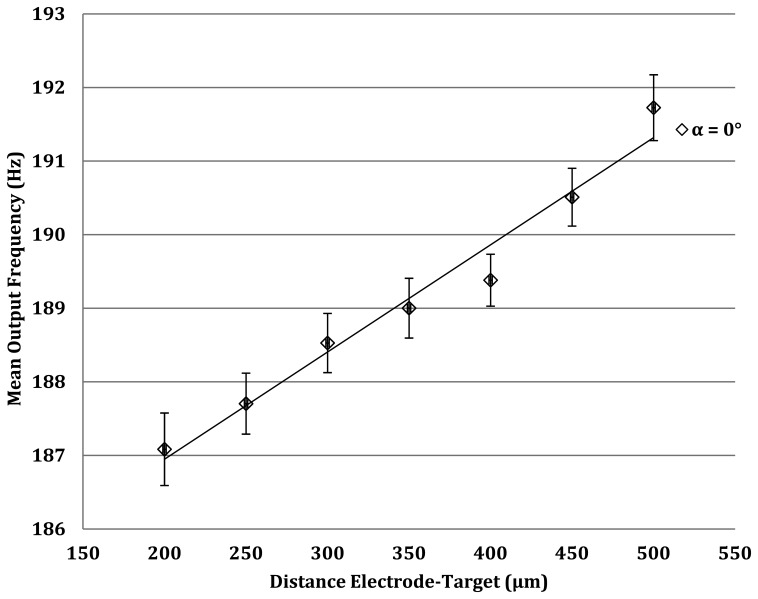
Output frequency as a function of electrode-target distance. Average curve on 5 measurements, with a standard deviation of 0.415 Hz.

The capacitance values range between 0.0177 pF and 0.0442 pF. Using Equation (3), the output frequency of the oscillator should thus be between 246 MHz and 251 Mhz. The parasitic plate capacitance is then connected to the circuit through a short coaxial cable (RG274U) that exhibits a capacitance of 1 pF. This figure, added to Cx, attenuates the impact of the measured capacitance on the output frequency. In this case, Cx thus varies between 1.017 pF and 1.044 pF, the resulting frequency ranging now being between 167 Mhz and 163 Mhz. Using a millimeter head, the coaxial probe of the oscillator is positioned at various distances from a non-conducting target and frequencies are recorded. Results are presented in Figure 10.

Measurement errors equal ±1%, which is the same order of magnitude as the previously seen optical method. The response time is however longer due to oscillator (±40 μs) and filter/buffer processing. Due to the electronics involved, this method is more complicated to implement than the optical method, but is easily miniaturized. Compared to the optical solutions, the quasi-linear behavior of the frequency with target distance is a major advantage in this design, and it provides similar detection accuracy with the advantage of compactness.

## 7. Comparison

Table 1 here below summarizes the comparison between the three techniques according to the following criteria: relative accuracy, miniaturization, implementation complexity, cost and innovation. It can be read that the resistive method is promising, despite an arguable precision for larger volume of liquids (up to 25% error in the worst case). This drawback is however limited to 10% for smaller volumes of liquids.

## 8. Conclusions/Outlook

An innovative microrobotic platform aimed at the microassembly of (sub)-millimeter components was designed. It is based on surface tension effects, which benefits from a powerful scaling law and provide useful compliance for microassembly purposea. Evaluation of the table position is however a limitation to assembly operation which had not been solved yet. In this paper, three methods were therefore investigated based on photo-sensing, capacitive, and resistance measurements. While photo-sensors and capacitive measurements are already well known, evaluating a micro-robotic table position using the electrical resistance of the droplets is very innovative, even if it is however only applicable to the gaseous environment. The three methods were tested and compared. Results show that the method based on droplets' resistance measurements is very promising. It is indeed compact, in comparison to photo-sensors, which cannot be miniaturized. It is simple to use and low cost, the only material needed being a voltmeter. The response is immediate in comparison to capacitive measurements, which have some response delay. Knowing the droplets' volume, the resistance measurements method is precise and repeatable. As deeply discussed in the paper, many parameters are involved in the physics of the droplet: the force F applied on it, the capillary pressure in the droplet, the volume of liquid, the droplet height and the electrical resistance. It has been shown by digital simulation and reference to previous experimental work that the droplet shape was driven by two parameters out of four: force, gap, volume of liquid, capillary pressure. Experimentally, we imposed the gap and the volume of liquid were imposed, so that the corresponding force and capillary pressure were dependent parameters. Obviously, the droplet geometry also determines the electrical resistance once the liquid conductivity is given. The major drawback of the method is that it is sensitive to liquid contamination and evaporation. However, since the timescale for evaporation/contamination may be longer than the timescale of a force variation, a perspective of this work is to be able to decouple this information. Consequently, we would recommend the resistive measurement method, compared to photo-sensors, because it is miniaturizable and, compared to the capacitive measurement method, simple to implement. In both cases, resistive measurement is also simpler, cheaper, and more precise.

## Figures and Tables

**Table 1.** Comparison of the three measurement methods.

	**Precision (Error)**	**Miniaturization**	**Easiness of Implementation**	**Cost**	**Innovation**
**Photo-sensing**	+ (5%)	− −	+ +	+	Well known
**Capactive method**	+ (1%)	+ +	−	− −	Well known
**Resistive method**	+ + (5–25%)	+ +	+	+ +	New
